# White matter tract-specific alterations in patients with primary restless legs syndrome

**DOI:** 10.1038/s41598-021-95238-6

**Published:** 2021-08-09

**Authors:** Hea Ree Park, Hye Ryun Kim, Seunghwan Oh, Joon-Kyung Seong, Eun Yeon Joo

**Affiliations:** 1 Department of Neurology, Inje University College of Medicine, Ilsan Paik Hospital, Goyang, Korea; 2 Global Health Technology Research Center, College of Health Science, Korea University, Seoul, Korea; 3 Department of Artifical Intelligence, Korea University, Seoul, Korea; 4 School of Biomedical Engineering, Korea University, 145, Anam-ro, Anam-dong 5-ga, Seongbuk-gu, Seoul, 02841 Republic of Korea; 5 Department of Neurology, Neuroscience Center, Samsung Medical Center, Sungkyunkwan University School of Medicine, 81 Irwon-ro, Gangnam-gu, Seoul, 06351 Republic of Korea; 6 Interdisciplinary Program in Precision Public Health, Korea University, Seoul, Republic of Korea

**Keywords:** Circadian rhythms and sleep, Neurological disorders

## Abstract

Prior diffusion tensor imaging (DTI) studies have investigated white matter (WM) changes in patients with primary restless legs syndrome (RLS), but the results were inconsistent. Here, we proposed using tract-specific statistical analysis (TSSA) to find alterations in specific WM tracts to clarify the pathophysiological mechanisms of RLS. We enrolled 30 patients with RLS and 31 age- and sex- matched controls who underwent brain magnetic resonance imaging, neuropsychological tests, and polysomnography. Fractional anisotropy (FA) maps obtained from whole-brain diffusion tensor imaging and TSSA were used to localize WM changes in patients with RLS. Subsequently, a comparison of FA values for each tract between patients and controls was performed. The associations between FA values and clinical, polysomnographic, and neuropsychological parameters in RLS patients were assessed. RLS patients demonstrated decreased FA values in the left corticospinal tract (CST) and cingulum, and in the right anterior thalamic radiation (ATR) and inferior fronto-occipital fasciculus (IFO). Patients’ attention/executive function and visual memory scores positively correlated with FA values in the right ATR, and anxiety levels negatively correlated with FA values in the right IFO. Additionally, the number of periodic leg movements and movement arousal index were negatively correlated with FA values in the left CST. The TSSA method identified previously unknown tract-specific alterations in patients with RLS and significant associations with distinct clinical manifestations of RLS.

## Introduction

Primary restless legs syndrome (RLS) is a sensorimotor disorder characterized by unpleasant sensations and an irresistible urge to move the legs, predominantly when resting or in the evening^1^. This condition commonly causes delayed sleep onset and sleep fragmentation; thus, patients with severe RLS suffer from chronic sleep loss, sometimes leading to adverse consequences in the daytime. Several studies to date have reported association between RLS and anxiety, depression, and impaired cognition^2–5^.

In the past few years, novel MRI modalities such as diffusion tensor imaging (DTI) and functional MRI (fMRI) have been used to identify structural or functional changes in the central nervous system of RLS patients that are undetectable by conventional MRI^6–15^. Among the known techniques, DTI is a noninvasive MRI method that estimates the microstructural integrity of white matter (WM) by measuring random movements of water within brain tissue^16^. Several DTI studies have assessed WM changes in patients with RLS, but the extent and features of WM abnormalities were inconsistent. Some DTI studies have observed disruptions to WM integrity close to the somatosensory cortex or prefrontal cortices, corpus callosum, internal capsule, basal ganglia, brain stem, spinal cord, and cerebellum^6,8–10,12^, while others did not find any differences in the WM of RLS patients compared with that of normal controls^7,11^.

Previous DTI studies of RLS have used the voxel-wise approach^6,8,10,12^ and tract-based spatial statistics (TBSS) method^7,9,17^. The voxel-wise approach can not specify anatomically which WM tracts are impaired, which is essential for identifying possible disruptions in connectivity between two specific brain regions. Moreover, it can be compromised by aligning multiple subjects into a standard space template such that interpretation of the result can be ambiguous^18^. The tract-based spatial statistics (TBSS) method, a widely used DTI technique, can facilitate investigations of local microstructural alterations through nonlinear registration followed by projection of diffusion parameters onto a WM skeleton^19^. However, this projection onto a group mean skeleton adopted in TBSS might mix differently oriented, multiple adjacent fibers given that TBSS discards fiber orientation^20^. Tract-specific statistical analysis (TSSA), a method that our group developed and validated^21^, improves the mapping of tract diffusion coefficients along the corresponding major anatomical tracts. This system uses the results of subject-specific tractography and a tract classification method that acquires the fiber directions in subject-specific tractography maps.

In the present study, we investigated tract-specific abnormalities in patients with RLS using the TSSA method to clarify the pathophysiological mechanisms of RLS. We then assessed whether such abnormalities were associated with various clinical presentations of the disease.

## Methods

### Participants

Thirty-four subjects older than 18 years of age who were diagnosed with primary RLS and 34 age- and sex- matched, healthy controls were enrolled in this study. The diagnosis of RLS was established by neurologists after an interview and physical examination in the sleep clinic based on updated international RLS study group diagnostic criteria^1^. We excluded subjects who had secondary RLS caused by anemia, pregnancy, gastrectomy, chronic kidney disease, or peripheral neuropathy. The severity of RLS symptoms was assessed using the International Restless Legs Scale (IRLS). Patient age at onset of RLS symptoms and duration of disease were recorded.

RLS patients and controls were excluded if they exhibited any of the following: (1) known sleep disorders other than RLS such as sleep disordered breathing, circadian sleep disorders, and parasomnia; (2) heart or respiratory disease; (3) history of cerebrovascular disease; (4) other neurological or psychiatric diseases; (5) alcohol or illicit drug abuse or current use of psychoactive medications; or (6) a structural lesion on brain MRI. All control subjects and 18 RLS patients who complained of frequent arousal during sleep or nonrestorative sleep underwent overnight polysomnography (PSG) to exclude other sleep disorders. Three RLS patients were excluded because PSG tests revealed undiagnosed obstructive sleep apnea (OSA), and two control subjects were excluded due to periodic leg movements (PLM index > 24). Finally, 31 RLS patients and 32 controls underwent neuropsychological tests and brain MRI. In this study, all of the participants were right-handed.

The Institutional Review Board of Samsung Medical Center approved the study protocol (IRB No. 2020-04-045-001) and informed consent was obtained from all subjects. This manuscript does not contain information or image that can lead to identification of a study participant. Study methods were carried out in accordance with approved guidelines.

### Neuropsychological assessments

Participants underwent a battery of neuropsychological tests in the following six broad domains: attention and executive function (digit span test, Corsi block tapping tests, trail-making tests A and B, and the Stroop test)^22–25^, verbal fluency (controlled oral word association test)^26^, verbal memory (Korean version of the California verbal learning test)^27,28^, visual memory (Rey complex figure test)^29^, and visuospatial function (Rey complex figure copy and Raven's colored progressive matrices tests)^30^. Composite scores and detailed information regarding these tests were described in a previous paper^31^. To examine emotional state, the Beck Depression Inventory (BDI) and Beck Anxiety Inventory (BAI) tests were administered^32^.

### Brain MRI acquisition

T1 and diffusion-weighted images were acquired from all 31 patients and 32 healthy controls using the same 3.0 Tesla MRI scanner (Philips 3.0 T Achieva). T1-weighted images were obtained using the following scanning variables: 0.5 mm sagittal slice thickness, over contiguous slices with 50% overlap, no gap, a repetition time (TR) of 9.9 ms, an echo time (TE) of 4.6 ms, a flip angle of 8° and a matrix size of 240 × 240 pixels. Images were reconstructed to 480 × 480 over a 240 mm field of view. In the whole-brain diffusion-weighted MRI examination, sets of axial diffusion-weighted single-shot echo-planar images were collected with the following parameters: 128 × 128 acquisition matrix, 1.72 × 1.72 × 2 mm^3^ voxels, 70 axial slices, a 220 × 220 mm^2^ field of view, a TE of 60 ms, a TR of 7385 ms, a flip angle of 90°, a slice gap of 0 mm, and a b-factor of 600 s mm^−2^. For baseline images without diffusion weighting (the reference volume), diffusion-weighted images were acquired from 45 different directions. All axial sections were acquired parallel to the anterior commissure-posterior commissure line. The method of brain MRI acquisition was identical with our group’s previous DTI studies^33,34^.

### Tract-specific statistical analysis (DTI processing)

**Figure 1:**
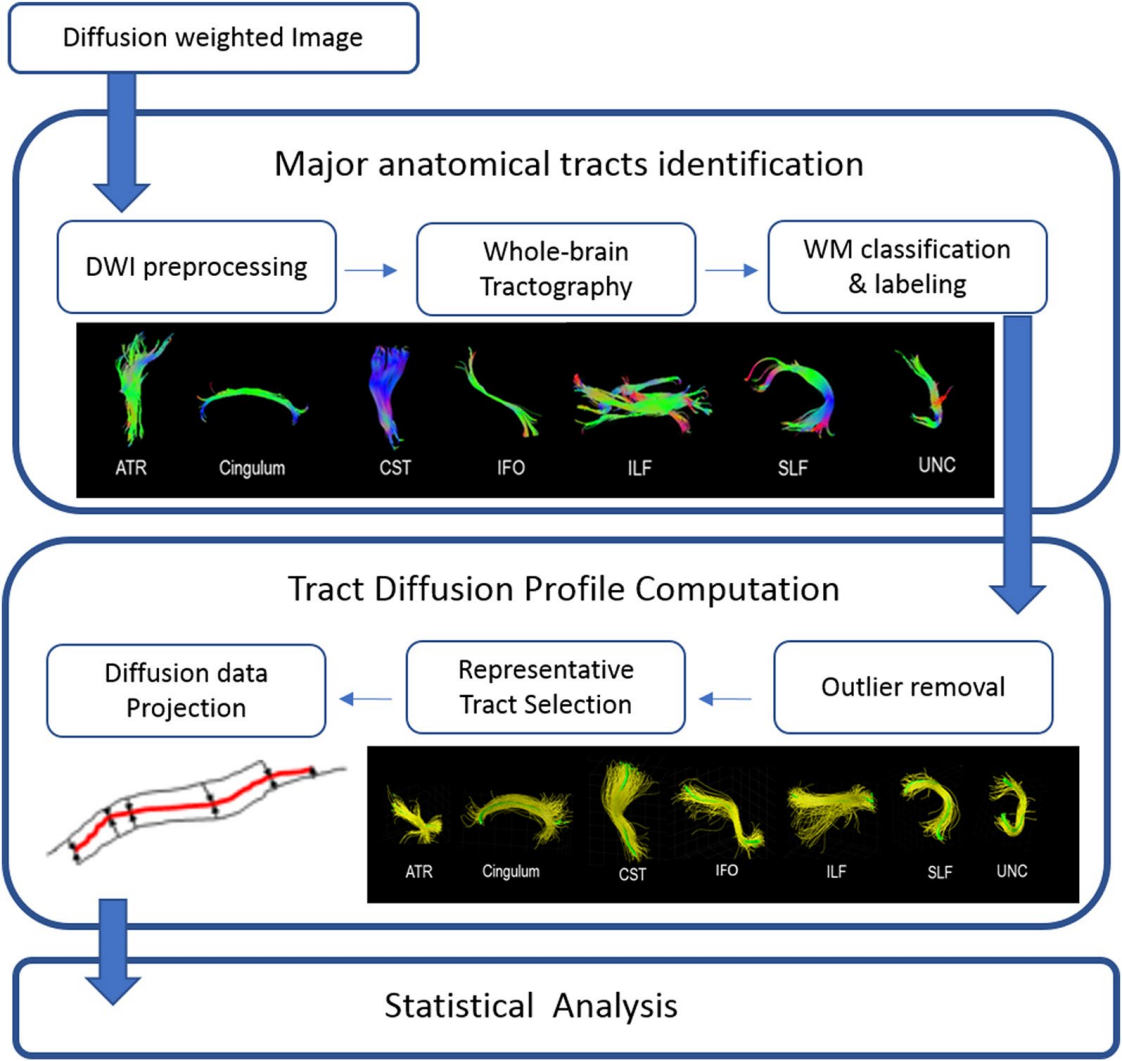
An overview of the white matter tract-based approach of the tract-specific statistical analysis (TSSA). DWI, diffusion weighted image; WM, white matter; ATR, anterior thalamic radiation; CST, corticospinal tract; IFO, inferior fronto-occipital fasciculus; ILF, inferior-longitudinal fasciculus; SLF, superior-longitudinal fasciculus; UNC, uncinate fasciculus.

TSSA consists of three steps of DTI preprocessing: (1) identifying the major anatomic tracts along the tractography derived fiber bundles after DTI preprocessing, (2) calculating a Tract Diffusion Profile, and (3) conducting statistical analysis. The detailed explanation of the TSSA method process was previously described^21,33,34^ and Fig. 1 presents an overview of the TSSA method.

First, diffusion-weighted images were preprocessed including skull stripping and eddy -current correction using the FMRIB software Library(FSL)^35^. Motion correction was performed by the affine alignment of the DWI volumes to the b0 image volume since b0 image is a reference volume for motion correction and it is not affected by eddy current distortions^36^. We applied deterministic tractography with DTI reconstruction using the Diffusion toolkit^37^. Deterministic tractography was processed based on Fiber Assignment by Continuous Tracking (FACT)^38^. Through this process, Fractional anisotropy (FA) values and whole-brain streamlines were obtained. Then, whole-brain streamlines were automatically classified and labeled into seven major tracts according to their shape and position, which was executed by our in-house software^39^. This method exploits expert-provided example data from multiple subjects. Example data contains multiple atlases from different subjects to consider the individual variability. Each atlas was constructed by automatically grouping whole-brain streamlines according to their shape similarity and manually labeling as seven anatomic bundles by experts. Multiple atlases are placed in the reference space. Given whole-brain streamlines of a test subject, they were transformed to the reference space of example through the registration between T1-weighted images. Then, the streamlines were clustered as tract group in the same way of constructing example data. Each tract group was automatically labeled by measuring the similarity with seven anatomical bundles in each atlas. The final label of each tract group was determined by voting with multiple example subjects. This method improved the accuracy of labeling relative to the region of interest (ROI)-based method^39^. The seven major tracts used were the anterior thalamic radiation (ATR), cingulum (CG), corticospinal tract (CST), inferior fronto-occipital fasciculus (IFO), inferior longitudinal fasciculus (ILF), superior longitudinal fasciculus (SLF), and uncinate fasciculus (UNC).

Next, we selected a representative streamline for each major tract for each subject following removal of streamline outliers. The outliers are streamlines which aren’t located in the regions specified by anatomical bundle definitions. Since a representative streamline should be long enough and located in the middle of the tract, a fiber density map was computed^21^. A fiber density map maintains the number of streamlines that pass through corresponding voxel in each voxel. The streamline with maximum density sum was chosen as a representative streamline.

Then, “Tract Diffusion Profile”^40^ was constructed by projecting FA values of all streamlines onto each representative tract. Since other streamlines didn’t have the same length and location with the representative streamline, point correspondence between a representative streamline and other streamlines was computed by the optimal point matching method^19^ that is robust to spatial distortion and FA value variability for curved fibers. The projected FA values for each major tract were averaged with the Mahalanobis distance as a weighting factor. For statistical analysis, a group-wise representative tract was further selected across subjects in the same way except with the constraint of accommodating more than 60% of subjects. The group-wise representative tract was resampled into 100 sample points using spline interpolation. As a final step, statistical analyses of FA values and clinical data such as patient-specific measures were performed. We considered FA the most suitable measurement to find WM alterations in RLS because RLS is a chronic neurological disorder related to disrupted sensorimotor neuronal transmission. To evaluate differences in FA values along each tract between RLS patients and controls, a permutation-based analysis of covariance, together with controlling for age and body mass index, was applied with 10,000 permutations. Since FA values in the tract profile were sampled at equidistant locations along the group-wise representative tract, a cluster-based statistics (CBS) method was used for multiple comparison correction^41,42^, which is often used in the neuroimaging field. F-value for the original set and the permutated set were computed by ANCOVA, which forms a null distribution of group difference. The CBS method corrects *p* with respect to number of sample points significantly clustered against another group. The number of sample points that have larger F-values than the given threshold was computed at first, which is cluster size. Then, the maximal size of clusters for each permutation which forms a null distribution of cluster size was calculated. The corrected p was estimated by the fraction of occurrences whose maximal size of clusters were not less than the cluster size of the original set. The threshold was 2.5.

To estimate the associations between FA values and clinical, polysomnographic, and neuropsychological parameters in the RLS group, permutation-based tests for correlation were performed before CBS. First, partial correlation coefficients were calculated for FA values along the representative tract, with the measures on clinical, polysomnographic, cognitive test, controlling age and sex as covariates. Like group comparison, the significance level of the correlation coefficient was adjusted by permutation testing with CBS that assumed a null distribution of maximum cluster extent^43^. The statistical analyses were conducted using MATLAB (Mathworks, Natick, MA, USA). CBS code is available at http://brein.korea.ac.kr/software/.

## Results

### Clinical characteristics

Finally, 30 patients and 31 controls were included in this analysis; one patient and one control group participant were excluded due to poor image quality. The demographics for all study participants are summarized in Table 1; participants were predominantly middle-aged and female. In RLS patients, the mean onset age for leg discomfort was 36.9 ± 14.1 years and the disease duration was 9.4 ± 8.5 years. Patients suffered from moderate to severe RLS-related symptoms (IRLS 29.9 ± 7.6). Thirteen (43.3%) RLS patients were taking dopamine agonists with or without alpha-2 delta ligands, and the duration of medical treatment was 2.6 ± 3.1 years. When comparing PSG tests, RLS patients (n = 15) demonstrated higher arousal index (17.6 ± 6.5 vs. 13.8 ± 5.3/h, *p* = 0.044), PLM index (16.2 ± 20.9 vs. 4.0 ± 5.8/h, *p* = 0.005), and movement arousal index (MAI 3.6 ± 4.3 vs. 1.0 ± 1.6/h, *p* = 0.005) relative to controls.

**Table1:** Demographics and sleep characteristics of patients with RLS and controls.

	Patients (n = 30)	Controls (n = 31)	*p*
Age	46.3 ± 13.0	44.1 ± 12.0	0.482
Men:women, no	8:22	8:23	0.939
Age at RLS onset (years)	36.9 ± 14.1		–
RLS duration (years)	9.4 ± 8.5		–
IRLS score	29.9 ± 7.6		–
Previous RLS medication use, n (%)	13 (43.3)		–
**Night polysomnography**	(n = 15)		
Time in bed (min)	446.0 ± 46.7	450.4 ± 43.2	0.767
Total sleep time (min)	389.4 ± 45.8	404.4 ± 43.3	0.305
Sleep latency (min)	12.5 ± 16.3	10.8 ± 10.9	0.676
Sleep efficiency (%)	87.4 ± 5.6	89.9 ± 5.8	0.180
Arousal index (h)	17.6 ± 6.5	13.8 ± 5.3	0.044*
WASO (min)	44.1 ± 22.5	35.1 ± 24.4	0.237
N1 sleep (%)	12.4 ± 6.3	11.9 ± 5.2	0.793
N2 sleep (%)	59.9 ± 11.4	56.8 ± 8.5	0.327
N3 sleep (%)	7.7 ± 5.1	8.5 ± 7.3	0.699
REM sleep (%)	20.1 ± 6.9	22.7 ± 4.2	0.189
AHI/h	4.0 ± 3.0	2.3 ± 3.0	0.084
PLMI (h)	16.2 ± 20.9	4.0 ± 5.8	0.005*
MAI (h)	3.6 ± 4.3	1.0 ± 1.6	0.005*

RLS, restless legs syndrome; IRLS, International Restless Legs Scale; WASO, wakefulness after sleep onset; REM, rapid eye movement; AHI, apnea–hypopnea index; PLMI, periodic leg movement index; MAI, movement arousal index.

*p < 0.05, independent t-tests for continuous variables and Fischer’s exact tests for categorical variables.

### Neuropsychological tests

RLS patients had lower scores for both forward and backward Corsi block-tapping (*p* = 0.005 and *p* = 0.028, respectively) compared with controls. The composite score for attention and executive functions was lower among RLS patients (*p* = 0.019). In addition, RLS patients had greater degrees of depression and anxiety than controls (*p* = 0.001 and *p* = 0.001, respectively). The results of the neuropsychological tests are summarized in Table 2.

**Table 2:** Neuropsychological test scores for patients with RLS and normal controls.

	Patients (n = 30)	Controls (n = 31)	*p*
**Attention and executive function composite score**	− 0.17 ± 0.66	0.23 ± 0.63	0.019*
Digit span, forward	8.5 ± 1.8	9.2 ± 1.9	0.147
Digit span, backward	7.1 ± 1.7	7.7 ± 1.6	0.152
Corsi block, forward	7.6 ± 1.5	8.8 ± 1.7	0.005*
Corsi block, backward	7.2 ± 1.4	8.1 ± 1.5	0.028*
Train making test B, time to completion	85.7 ± 39	75.6 ± 30.3	0.281
Stroop tests, correct responses	104.2 ± 12.1	105.0 ± 11.1	0.786
**Verbal fluency composite score**	− 0.11 ± 0.82	0.13 ± 0.92	0.295
COWAT, phonemic	33.2 ± 10.8	39.0 ± 12.1	0.058
COWAT, semantic	40.7 ± 9.3	40.7 ± 10.8	0.990
**Verbal memory composite score**	− 0.13 ± 0.77	0.13 ± 0.95	0.264
CVLT, total	53.4 ± 7.0	57.9 ± 9.8	0.060
CVLT, short	11.3 ± 2.6	12.1 ± 2.8	0.267
CVLT, long	12.2 ± 2.1	12.9 ± 2.4	0.271
CVLT, recognition	15.0 ± 1.2	15.0 ± 1.3	0.835
**Visual memory composite score**	0.09 ± 0.73	− 0.06 ± 0.96	0.885
RCFT, immediate	22.0 ± 5.3	21.9 ± 6.5	0.974
RCFT, delayed	21.4 ± 5.2	21.4 ± 6.2	0.964
RCFT, recognition	20.6 ± 1.8	20.3 ± 2.5	0.715
**Visuospatial function composite score**	0.04 ± 0.91	− 0.06 ± 0.97	0.948
RCFT, copy	34.7 ± 1.5	34.6 ± 1.7	0.774
RCPM	32.3 4.4	33.0 ± 2.8	0.530
**Beck Depression Inventory**	13.3 ± 7.9	6.3 ± 6.6	0.001*
**Beck Anxiety Inventory**	8.0 ± 5.4	3.5 ± 4.0	0.001*

RLS, restless legs syndrome; COWAT, controlled oral word association test; CVLT, California verbal learning test; RCFT, Rey complex figure test; RCPM, Raven's colored progressive matrices test.

*p < 0.05; independent t-tests.

### Comparison of TSSA results between patients with RLS and the control group

**Figure 2:**
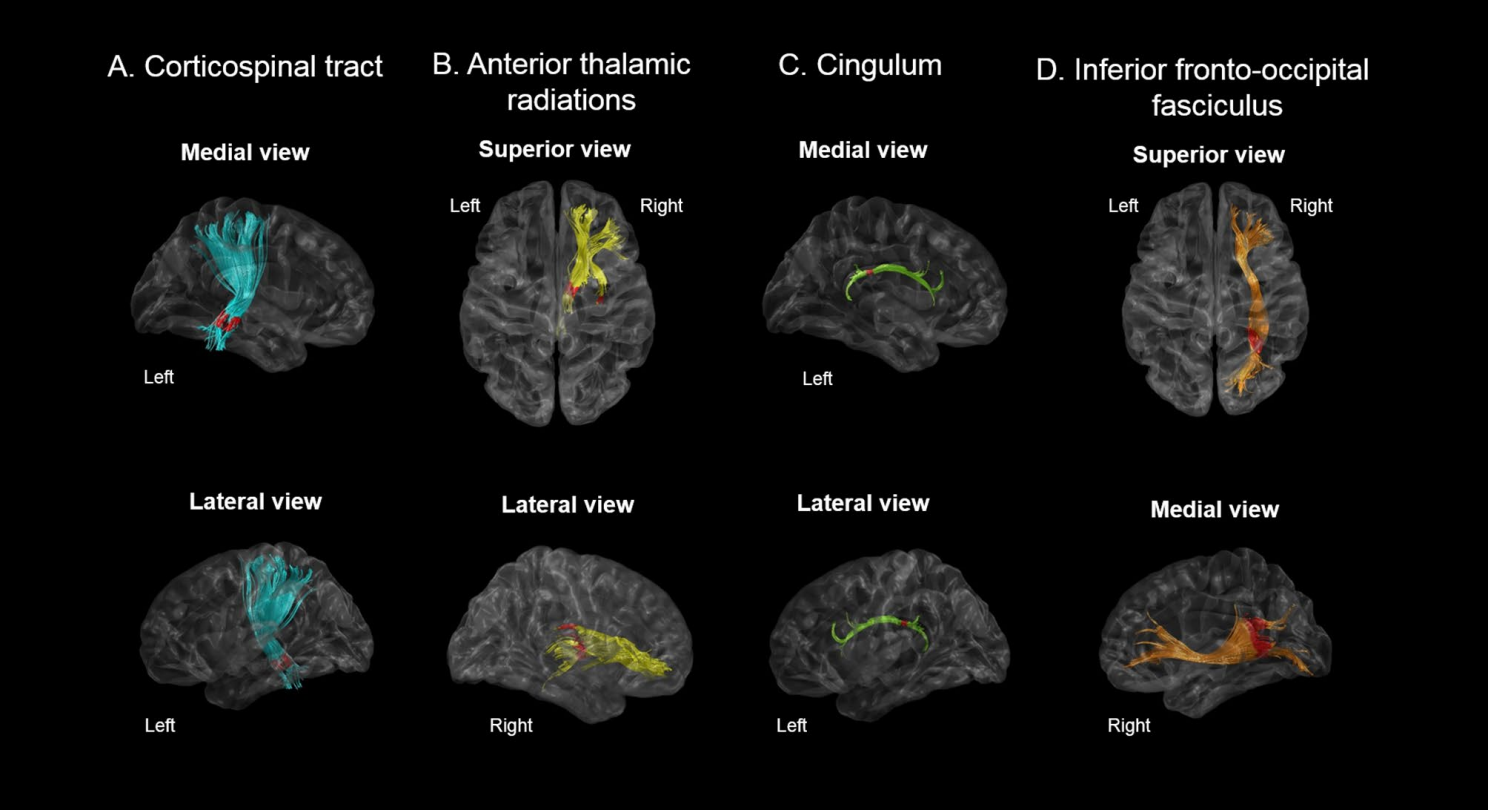
TSSA results revealed different FA values between RLS patients and control groups for (**A**) the left corticospinal tract, (**B**) the right anterior thalamic radiations (**C**) the left cingulum, and (**D**) the right inferior fronto-occipital fasciculus. Red colors indicate the portions of fiber tracts where FA values significantly reduced in RLS group compared to controls (*p* < 0.05). TSSA, tract-specific statistical analysis; FA, fractional anisotropy.

RLS patients had significantly lower FA values in the inferior part of the left CST (*p* = 0.018), the left CG close to the posterior cingulate cortex (*p* = 0.010), the posterior part of the right ATR (*p* = 0.013), and the posterior portions of the right IFO (*p* = 0.008) compared with controls. A detailed description of FA values according to the noted percentage of fiber length and portion of the fiber tract with significant FA differences is shown in Fig. 2 and Supplementary Figure 1.

### Association between TSSA values and clinical, cognitive, and polysomnographic parameters

**Figure 3:**
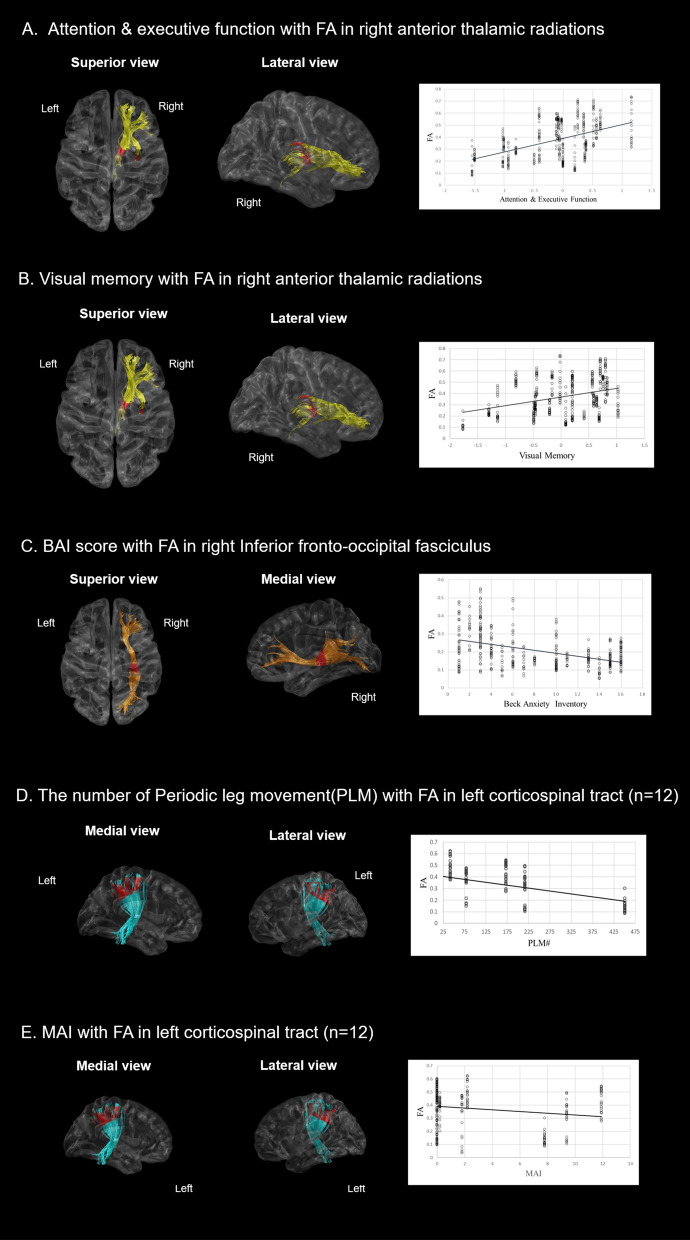
Correlation analysis with adjustments for age and gender between FA values and neuropsychological/PSG indices in the RLS group. (**A**,**B**) FA values in the right anterior thalamic radiation were significantly associated with the composite score of attention/executive function, and visual memory. (**C**) FA values in the right inferior fronto-occipital fasciculus were negatively correlated with BAI. (**D**,**E**) FA values in the left corticospinal tract were negatively correlated with number of PLMs and MAI. Red colors indicate the portions of fiber tracts where FA values significantly correlated with neuropsychological/PSG indices. FA, fractional anisotropy; PSG, polysomnography; RLS, restless legs syndrome, BAI, Beck anxiety inventory; PLM, periodic leg movement; MAI, movement arousal index.

For the relationships between tract-specific FA values and neuropsychological test results, FA values in the posterior part of the right ATR were positively correlated with composite scores for attention/executive function (*p* = 0.028, Fig. 3A) and visual memory (*p* = 0.042, Fig. 3B). FA values in the posterior part of the right IFO were negatively correlated with BAI (*p* = 0.038, Fig. 3C). Patients with PLMs during sleep as seen during PSG (n = 12) had negative correlations between FA values in the subcortical part of the left CST and number of PLMs (*p* = 0.044, Fig. 3D) and MAI (*p* = 0.045, Fig. 3E).

## Discussion

In this cross-sectional, case–control study, the TSSA method revealed that patients with RLS had altered FA values in multiple major WM tracts, correlated with neuropsychological and polysomnographic parameters. More specifically, patients with RLS had significantly decreased FA values in the left CST, ATR, and CG and in the right IFO, suggesting disrupted WM integrity in these tracts. Furthermore, the FA values in the left CST and right IFO in RLS were correlated with PLMs and anxiety, respectively, and FA values in the right ATR were associated with attention/executive function and visual memory of RLS patients.

Several DTI studies of RLS reported alterations of motor and somatosensory systems, but the details of the results have been different among studies. Some studies reported decreased FA values in variable supratentorial WM tracts including WM close to the motor and somatosensory cortices, CG, putamen, genu of the corpus callosum, and internal capsule^6,8,12^. Two studies reported involvement of infratentorial structures such as brain stem, cerebellum, and upper cervical cords in RLS patients^9,10^. In contrast, studies by Rizzo^7^ and Zhuo^11^ did not demonstrate any alteration in WM in RLS patients^7,11^. The discrepancies between results might be explained by differences in post-processing methodologies between studies and the technical limitations of these previous methods. These studies used voxel-based approaches^6,8–10,12^ or the TBSS method^7,11^, which resulted in mixed values for multiple fiber tracts with different orientations. Differences in sample size, magnetic field strength, and rate of possible comorbid sleep disorders also might have contributed to the discrepancies between results. Furthermore, most previous studies excluded patients with other sleep disorders based solely on patient interviews without verifying the validity of their reports by PSG. Undiagnosed comorbid sleep disorders such as OSA might have influenced the DTI results in these studies.

This study has merits compared with previous studies. The most obvious strength of the study is its adoption of a tract-specific approach, TSSA, to identify local deficits more precisely in anatomical WM tracts. This TSSA method enabled us to identify local disruptions in specific fiber tracts using the orientation of all fiber tracts unlike VBM and TBSS. TBSS captures regional microstructural alterations based on an FA skeleton in a voxel-based coordinate map. Previous studies, which concentrated on ‘exact location’ of DTI abnormalities in RLS patients, did not specify which white matter tracts are impaired^6–12^. The present research focused on ‘specific tract’ rather than ‘specific location’ because we thought that specification of involved WM tracts would be more meaningful than specific location to clarify functional abnormalities. In fact, similar to TSSA, tract profile based methods such as Automated Fiber Quantification from Vistasoft can also consider local disruptions in specific fiber tract^40^. However, TSSA method employs a multiple atlases-based WM tract-classification method without using predefined ROIs/atlases to mask streamlines and optimal point matching method^19^ for the correspondence between representative streamline and streamlines in order to utilize the full tract length. Using multiple atlases from different subjects enables us to reflect larger individual variability of bundle shapes and trajectories than single atlas-based methods^44,45^. Therefore, it is not limited by intersubject variability that may enable more accurate localization of WM changes by individual. This process demonstrated its clinical utility as it identified tract-specific abnormalities in patients with cognitive impairment and in those with other sleep disorders such as narcolepsy and OSA^21,33,34^. Another strength of this study is that participants who suffered from frequent arousal or nonrestorative sleep underwent PSG for exclusion of undiagnosed comorbid sleep disorders, to minimize confounding variables. 3.0 T MRI scans also increased the reliability of results reported in this study.

The CST is a major neural tract known to convey sensorimotor information^46^. We found that FA values in the brain stem part of the left CST were lower in RLS patients than in controls. These results are in line with previous VBM studies that demonstrated decreased GM and WM volumes in the primary sensorimotor cortex^47,48^ and with DTI studies that reported alteration of WM integrity near the motor and sensory cortices and the IC portion of the fronto-pontine tract^6,8,12^. As sensorimotor symptoms are the main manifestations of RLS, it is tempting to assume that FA reduction in the CST might provoke abnormal neuronal transmission of sensorimotor output. The FA values in the CST were significantly correlated with PLMs, a common motor symptom of RLS, which supports this postulation.

This study also suggested that disruptions in specific tracts were associated with cognitive impairments and psychiatric problems in patients with RLS. We observed decreased FA values in the right ATR and IFO and the left CG of RLS patients with mood instability and impaired attention/executive function. The FA values in the right ATR were negatively correlated with attention/executive function and visual memory, which means decreased FA values in the right ATR were associated with impaired cognitive function of these domains. Impaired prefrontal function is the most well-known cognitive manifestation in RLS patients. Although there was no significant difference in memory function between RLS patients and controls in this study, such memory dysfunction has been reported in previous studies^49,50^. Moreover, microstructural alterations in the CG are associated with frontal lobe dysfunctions in multiple neuropsychological diseases such as mild cognitive impairment and schizophrenia^51,52^. Concerning psychiatric aspects, decreased FA values in the right IFO were associated with anxiety in RLS patients. The IFO is the longest associated bundle, and the IFO deep layer is suggested to be associated with numerous neuropsychiatric functions^27,53^. Therefore, it can be surmised that altered integrity in these tracts plays a role in the impaired cognitive performance and psychiatric comorbidities seen in RLS patients.

This study’s limitations should be addressed. First, a history of dopaminergic medications or other medications for RLS that could affect WM integrity were not considered as covariates in our statistical analyses. Second, the timing of MRI was not consistent among participants, which could be a confounding factor as the abnormal sensations reported in RLS patients commonly occur or worsen in the evening. However, the effects of medication and the diurnal variation in symptoms of RLS with respect to DTI would be far less than those seen in other dynamic imaging modalities such as fMRI and perfusion MRI. Third, seven major tracts were investigated in this study because of the restriction of example data in tract segmentation method. The other WM tracts could be considered in the future study with other tract segmentation method^54–56^. In addition, we focus on FA values since the meaning of abnormalities in other measures has not been fully understood in patients with RLS. Other metric such as MD, RD or tractometry combining multiple measures could be investigated in the future study^57–59^. Lastly, the study’s relatively small sample size may limit its statistical power. Instead, we minimized confounding factors by excluding subjects whose comorbid sleep disorders were undiagnosed using PSG screening.

To our knowledge, this is the first DTI study to adopt the TSSA method to explore tract-specific abnormalities in patients with RLS. This novel technique enabled us to identify alterations in specific WM tracts in RLS patients, in contrast with findings reported in previous DTI studies that focused on brain region abnormalities related to RLS. Patients with RLS demonstrated impaired WM integrity in the left CST and CG, and in the right ATR and IFO, which was associated with cognitive performance, anxiety, and PLMs. This study contributes to a deeper understanding of RLS manifestations due to the altered integrity apparent in WM tracts. This study provides new insights into the pathophysiological mechanisms of primary RLS.

## Supplementary Information

Supplementary Information 1.

Supplementary Information 2.

## Data Availability

The datasets generated during and/or analyzed during the current study are available from the corresponding author on reasonable request.
