# Supplementary material for: White matter tract-specific alterations in patients with primary restless legs syndrome
Source: Sci Rep. 2021 Aug 9;11:16116. doi: 10.1038/s41598-021-95238-6 (PMC8352949; doi:10.1038/s41598-021-95238-6)
Supplement: Supplementary file 1 — Supplementary Information 1. [file 41598_2021_95238_MOESM1_ESM.docx]

**Supplementary figure 1.** Distribution of FA values between RLS patients and control groups for the (A) left corticospinal tract, (B) the right anterior thalamic radiations (C) the left cingulum, and (D) the right inferior fronto-occipital fasciculus. Black line indicates the portions of fiber tracts where FA values significantly reduced in RLS group compared to controls (*p*<0.05).
