# Supplementary figures and images for: White matter tract-specific alterations in patients with primary restless legs syndrome
Source: Sci Rep. 2021 Aug 9;11:16116. doi: 10.1038/s41598-021-95238-6 (PMC8352949; doi:10.1038/s41598-021-95238-6)

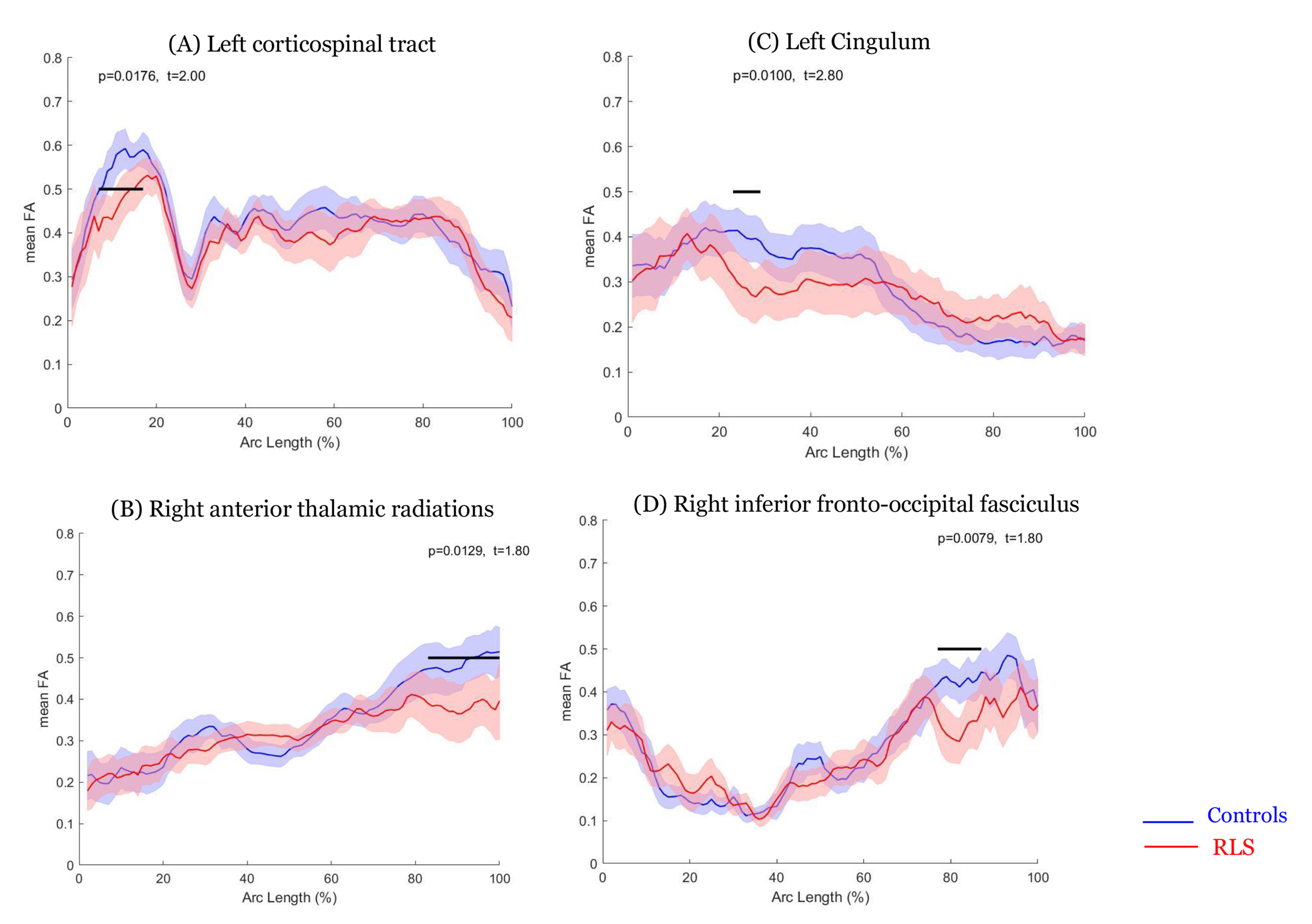

Supplement: Supplementary file 2 — Supplementary Information 2. [file 41598_2021_95238_MOESM2_ESM.png]
